# Being treated in higher volume hospitals leads to longer progression-free survival for epithelial ovarian carcinoma patients in the Rhone-Alpes region of France

**DOI:** 10.1186/s12913-017-2802-2

**Published:** 2018-01-04

**Authors:** Marius Huguet, Lionel Perrier, Olivia Bally, David Benayoun, Pierre De Saint Hilaire, Dominique Beal Ardisson, Magali Morelle, Nathalie Havet, Xavier Joutard, Pierre Meeus, Philippe Gabelle, Jocelyne Provençal, Céline Chauleur, Olivier Glehen, Amandine Charreton, Fadila Farsi, Isabelle Ray-Coquard

**Affiliations:** 1 Univ Lyon, University Lumière Lyon 2, GATE L-SE UMR 5824, 93 Chemin des Mouilles, F-69130 Ecully, France; 2 Univ Lyon, Léon Bérard Cancer Center, GATE L-SE UMR 5824, F-69008 Lyon, France; 3 Leon Berard Cancer Centre, Lyon, France; 4 University Hospital of Lyon, Lyon, France; 5 Private Hospital Jean Mermoz, Lyon, France; 6 Univ Lyon, University Claude Bernard Lyon 1, ISFA, Laboratoire SAF, F-69007 Lyon, France; 7 Lest-UMR 7317, Aix-Marseille University, Marseille, France; 8 Groupe Hospitalier Mutualiste, Grenoble, France; 9 Hospital Métropole de Savoie, Chambery, France; 10 University Hospital, Saint-Priest-en-Jarez, France; 11 Réseau Espace Santé Cancer Rhône-Alpes, Lyon, France; 12 Univ Lyon, Léon Bérard Cancer Center, EA 7425 HESPER, F-69008 Lyon, France

**Keywords:** Disease management program, France, Epithelial ovarian cancer, Propensity score, Centralization of care

## Abstract

**Background:**

To investigate the relationship between hospital volume activities and the survival for Epithelial Ovarian Carcinoma (EOC) patients in France.

**Methods:**

This retrospective study using prospectively implemented databases was conducted on an exhaustive cohort of 267 patients undergoing first-line therapy during 2012 in the Rhone-Alpes Region of France. We compared Progression-Free Survival for Epithelial Ovarian Carcinoma patients receiving first-line therapy in high- (i.e. ≥ 12 cases/year) vs. low-volume hospitals. To control for selection bias, multivariate analysis and propensity scores were used. An adjusted Kaplan-Meier estimator and a univariate Cox model weighted by the propensity score were applied.

**Results:**

Patients treated in the low-volume hospitals had a probability of relapse (including death) that was almost two times (i.e. 1.94) higher than for patients treated in the high-volume hospitals (*p* < 0.001).

**Conclusion:**

To our knowledge, this is the first study conducted in this setting in France. As reported in other countries, there was a significant positive association between greater volume of hospital care for EOC and patient survival. Other factors may also be important such as the quality of the surgical resection.

**Electronic supplementary material:**

The online version of this article (10.1186/s12913-017-2802-2) contains supplementary material, which is available to authorized users.

## Background

While epithelial ovarian carcinoma (EOC) is known to be a serious disease, its impact is often underestimated due to its relatively low incidence and its high mortality rate. EOC remains the eighth most common cancer for women, with an incidence rate of 11 to 12 women for every 100,000 women/year. EOC remains the main cause of gynecological cancer deaths in industrialized countries, with a mortality rate in France of about 4/100000 persons per year [1]. Indeed, survival estimates, based on the FRANCIM network registry data between 1989 and 2004, indicate an overall survival rate at 5 and 10 years of 37% and 28%, respectively. Relapse-free survival and overall survival of patients are related to the characteristics of the disease, the patient herself, and the disease management. The latter is based on surgery with a complete tumor resection, which can have a significant impact even on stage IV disease. Optimal debulking also has a positive impact on outcomes, but far less so than complete tumor resection. Surgical debulking has a positive impact on all histological subgroups. Nevertheless, mucinous carcinoma remains a strong independent prognostic factor for the disease [2]. Several retrospective studies have investigated the relationship between outcomes of ovarian cancer treatment and the type of care provider [3]. A higher quality of surgery when performed by gynecological oncologists in specialized hospitals (i.e. referral centers) and only small differences in chemotherapy regimens have been reported between the settings. Some studies have also investigated the impact of the centralization of care, in terms of volumes and patient outcomes [4–14]. Patients are more likely to be optimally debulked in a high-volume hospital or by a specialized provider. These studies have also shown that patients have better survival outcomes in high-volume hospitals. However, most of these studies focused on advanced stage disease, and none were carried out in France. The majority of patients with ovarian cancer do not receive care in specialized settings [8]. Moreover, there is still substantial national debate about the necessity of centralization of care for ovarian cancer, with major economic implications that need to be assessed.

As stipulated by the French ministerial order of 27 March 2007, French legislation requires a minimum hospital volume activity in order to receive authorization to treat gynecological cancers [15]. Thus, a hospital needs to perform more than 20 surgeries per year for gynecological cancers, such as cervical, ovarian, vaginal, uterine, and vulvar cancers, to receive authorization to treat patients with these specific diseases the following year. Patients are free to choose at which of the hospitals authorized to treat gynecological cancers they will be treated. Moreover, ovarian cancer is considered to be a Long Duration Disease (LDD) by the French social security system. Therefore, in this setting, 100% of the treatment costs are reimbursed by the government, based on the reference cost set by the social security system. However, patients may nonetheless incur additional fees, most often in private for-profit hospitals. For patients with a supplementary health insurance (already 95% of French residents were covered even before the reform of 2016) additional fees may be partially or fully reimbursed by their supplementary health insurance, depending on the type of policy that they have selected.

The aim of this study was to compare Progression-Free Survival (PFS) with first-line therapy for EOC patients treated in high- versus low-volume hospitals in the Rhone-Alpes region of France in 2012. The novelty of this study lies in part with the use a sophisticated statistical analysis that allows for proper control of the strong selection bias between patients treated in high- versus low-volume hospitals.

## Methods

### Patient population and study design

This retrospective study using a prospectively implemented database was conducted on an exhaustive cohort of patients treated in first-line during 2012 in the Rhone-Alpes Region of France.^1^ The database was constructed by the EMS team (Medical Evaluation and Sarcomas) from the Leon Berard cancer research center (Lyon, France). They established an exhaustive list of all patients newly diagnosed with ovarian cancer in the region using existing lists from oncology treatment-coordinated centers (3C), and from pathologists in the region. The inclusion criteria were: first-line treatment for EOC, diagnosed in 2012, an incident case, more than 18 years of age, residing in France, and being treated in a hospital in the Rhone-Alpes region. The exclusion criteria were: non-epithelial disease, relapsed disease, less than 18 years of age, or patients living in the region who had undergone treatment in another region of France. Finally, Clinical Research Assistants from the EMS team collected the data at all of the included hospitals, 2 years after diagnosis period. For each patient, their age, cancer history (yes or no), presence of ascites (yes or no), histology (e.g. high-grade serous carcinoma, low-grade serous carcinoma, mucinous, endometrioid, clear-cell, or unknown), FIGO stage (I to IV), tumor grade (1 to 3), residual tumor (CC0: no residual; CC1 or CC2: microscopic or macroscopic residual), reoperated (yes or no), and the type of chemotherapy (e.g. neoadjuvant, adjuvant, both, or none) were recorded as well as the dates of progression and/or death or last contact.

### Statistical methods

Progression-Free Survival (PFS) was defined as the time elapsed between the diagnosis and disease progression (loco-regional or metastatic) or death from any cause. To determine whether the PFS was longer in high-volume hospitals (HVH), we needed to define a threshold based on the volume of activity of hospitals in the study. The upper quartile was chosen as the cut-off value for HVH where 25% of EOC patients in first-line treatment during the year 2012 are categorized as being treated in HVH versus 75% as being treated in Low-Volume Hospitals (LVH). As a sensitivity analysis, we also considered two other thresholds using the lower quartile and the median of the volume activity, in order to get two groups of patients treated as 75% in HVH-25% in LVH and 50% treated in HVH-50% in LVH, respectively. Investigation of whether there are differences in survival according to the volume activity of hospitals requires controlling for differences between the two groups of patients (i.e. those treated in HVH vs. LVH). Indeed, without randomization, patients in high- and low-volume facilities may be different in regard to observed or unobserved factors that could affect outcomes [16]. Since the database contained an abundance of patient characteristics, we relied on methods that adjust for observable selection bias (i.e. multivariate analysis and propensity score methods). In all statistical analysis, we relied on a 5% level of significance. Patients for whom the hospital for the first-line treatment or for which all of the characteristics were missing were excluded from the analysis.

### Multivariate analysis

A common approach when dealing with confounding factors is to use multivariate regression [17]. The principle was to regress the survival time on an indicator variable denoting HVH or LVH, and to control for prognostic factors such as age, histology, FIGO stage, grade, neoadjuvant chemotherapy, cancer history, and the presence of ascites. This specification was replicated for the three different thresholds that we used to define a HVH. In practice, we first ran a Cox proportional hazard model of the Progression-Free Survival (PFS) on the set of covariates, and we then tested whether the hazard was proportional or not by the Schoenfeld residual test and with a Log-Log plot [18]. Then, if the proportional hazard assumption was upheld, the preferred model was a semiparametric Cox proportional hazard regression. If not, we resorted to a parametric determination with an Accelerated Failure Time (AFT) model. With the AFT model, we had to choose a parametric distribution of the hazard. A common practice was to at first determine a Generalized Gamma model which includes the Exponential (*k*  = *σ*  =  1), Weibull (*k*  =  1), Lognormal (*k* = 0), and Gamma (*σ* =  1) distributions. It was then possible to test for these parameters in order to choose between these distributions by a likelihood ratio test.

Multivariate analysis only allowed for determination of a relative effect, which could be seen as a conditional treatment effect: the average effect of being treated in a higher volume hospital at the individual level, as if a patient in a low-volume hospital was treated in a higher volume hospital. Propensity score methods had the advantage of allowing determination of both absolute and relative treatment effects, as the CONSORT statement recommends evaluation of the treatment effect in an observational study [16].

### Propensity score matching using inverse probability weighting

Propensity score methods were also applied to control for the selection bias and to determine both a relative and an absolute treatment effect. These two effects could be seen as marginal treatment effects in the sense that they corresponded to the difference in outcomes between the groups of patients in high- versus low-volume hospitals [16]. By comparison, multivariate analysis allowed for evaluation of a conditional effect and not a marginal effect. In practice, we used Inverse Probability Weighting (IPW) using the propensity score. We used the standardized difference in means instead of the t-test to compare the baseline characteristics, as recommended by Austin [16] and Stuart [19].

The IPW method balances out the covariate of the two groups by weighting all patients in the data base by the inverse of their propensity score. The propensity score was the conditional probability for a patient to be treated in a high-volume hospital, conditionally to observables characteristics. We determined this probability by fitting a logit model of an indicator variable denoting high- or low-volume hospitals on age, histology, FIGO stage, grade, cancer history, neoadjuvant chemotherapy, and the presence of ascites. We excluded predictive variables of outcomes that may depend on patient choice and subsequent interventions from this model, and we only controlled for patient characteristics at the time of diagnosis (i.e. prior to the patients receiving their first-line treatment). Again, the determination of the weights was performed for each threshold of the hospital volume activities.

We used the stabilized weights of the IPW proposed by Robins [20]. It should be note that *T*_*i*_ is the treatment variable, *p*_*i*_ the propensity score, and *f* (*T*) the distribution of the treatment which was determined by a logit model without considering covariates. In order to determine the Average Treatment effect on the Treated (ATT), weights can be calculated with the formula in eq. (1).

1 $$ {w}_i^{ATT}=f{(T)}^{\ast}\left[{T}_i+\frac{p_i\left(1-{T}_i\right)}{\left(1-{p}_i\right)}\right] $$

An Adjusted Kaplan-Meier Estimator (AKME), as proposed by Xie and Liu [21] and a univariate Cox model in the weighted sample, as described by Cole and Hernan [22], were then applied in order to determine the absolute and relative impact, respectively, of the concentration of care on the survival of EOC patients. We used the robust variance estimator of Lin and Wei [23] for the weighted Cox model, to take into account the within matched set correlation due to the matching process. In order to test for a significant difference in survival curves for the two groups, we used the adjusted log rank test as proposed by Xie and Liu [21], to take into account that patients in high- and in low-volume hospitals are no longer independent after weighting using the IPW.

Statistical analyses were conducted using STATA version 14.0 software (Stata Corp, College Station, TX) and R Statistical Software version 3.4.0 (Foundation for Statistical Computing, Vienna, Austria).

## Results

### Patient and hospital characteristics

**Fig. 1:**
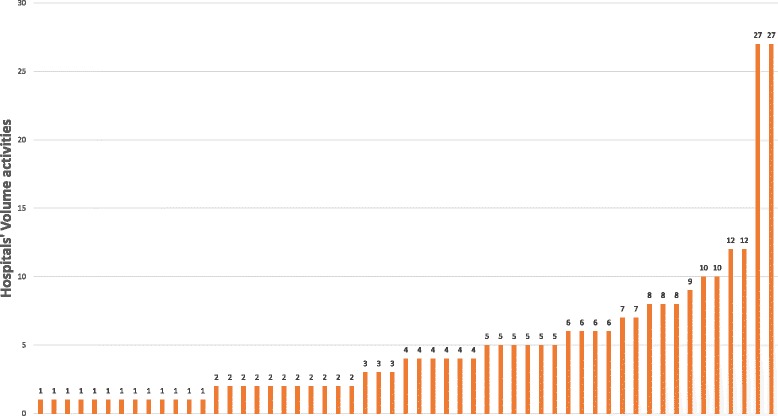
Distribution of annual hospital volume activities

In 2012, 267 patients were identified with an EOC in the Rhone-Alpes region, although only 231 (87%) were used in the modeling due to missing data. Patients were treated in 55 different hospitals across the entire region, including 51 low-volume hospitals (i.e. volume < 12 cases/year) and 4 high-volume hospitals. The median volume activity by hospital for the HVH was 19.5 (from 12 to 27) patients treated for EOC per year, versus 3 (from 1 to 10) for the LVH. Figure 1 depicts the distribution of hospital volume activities. In this figure, each bar represents a specific hospital. The distribution varied among the hospitals, from a minimum of one patient to a maximum of 27 patients in 2012.

Thirteen of the 55 facilities had treated only one patient in 2012 (24%), and 24 had treated no more than two patients (40%). The higher volume hospitals were either university hospitals, cancer centers, or private hospitals. Only 78 patients (37%) in a first-line setting were treated in a high-volume hospital. Of the 231 total subjects, 131 patients relapsed and 89 patients died (with or without having relapsed) during the follow-up. Table 1 lists the patient characteristics, comparing patients treated in high- versus low-volume hospitals. At baseline (i.e. before matching), the higher volume hospitals tended to treat a higher proportion of grade 3 tumor patients (*p* = 0.006) and a lower proportion of grade 1 tumor patients (*p* = 0.019), compared to lower volume hospitals. The HVH also tended to treat a lower proportion of stage I patients (*p* = 0.026), and a higher proportion of stage III patients (*p* = 0.046). It can be seen that 81% of the patients treated in the HVH were Grade 3 versus only 63% of the patients treated in the LVH. There was a significantly higher proportion of patients with no residual tumor (CC0) (*p* < 0.001) and a significantly lower proportion of reoperation (*p* < 0.001) in higher volume hospitals. Patients in lower volume hospitals were more often treated with a post-surgery chemotherapy only (*p* = 0.047)), while patients in higher volume hospitals were more likely to be treated with both a neoadjuvant and a post-surgery chemotherapy (*p* < 0.001). These differences in the use of chemotherapy are only from a descriptive point of view, and do not take into account that the HVH were treating patients with a higher tumor stage and grade.

**Table 1:** Patient characteristics at baseline (threshold of 12 patients)

	Low-volume hospital (*n* = 78 patients)	High-volume hospital (*n* = 189 patients)		
	m	m	*P*-value	%bias
Age	63.78	66.10	0.193	17.9
Cancer history	0.14	0.17	0.622	6.6
Ascites	0.60	0.69	0.148	19.7
Histology:				
- HGSC	0.20	0.62	0.142	20.4
- LGSC	0.06	0.04	0.443	−11.0
- Mucinous	0.10	0.03	0.048	−30.2
- Endometrioid	0.14	0.13	0.867	−2.3
- Clear cell	0.06	0.04	0.421	−11.4
- Unknown	0.11	0.15	0.447	10.2
FIGO Stage:				
- I	0.25	0.13	0.026	−31.8
- II	0.05	0.08	0.438	10.1
- III	0.56	0.69	0.046	27.5
- IV	0.14	0.10	0.458	−10.3
Tumor Grade:				
- 1	0.16	0.05	0.019	−35.8
- 2	0.20	0.14	0.216	−18.0
- 3	0.63	0.81	0.006	40.3
Chemotherapy:				
- Neoadjuvant only	0.17	0.12	0.228	−16.8
- Post-surgery only	0.47	0.33	0.047	−27.1
- Neoadjuvant & post-surgery	0.18	0.45	0.001	60.2
- None	0.18	0.10	0.115	−22.3
Reoperation	0.34	0.12	0.001	−54.1
No residual disease after debulking surgery	0.70	0.45	0.001	50.5
			*Mean:*	*24.3*
			*Median:*	*20.1*

m: mean (frequency) if the covariate is continuous (binary) / % bias, also known as the standardized difference of the mean

*HGSC* High-Grade Serous Carcinoma, *LGSC* Low-Grade Serous Carcinoma

### Multivariate analysis

The Schoenfeld residual test revealed that the null hypothesis of proportional hazard was not rejected (*p* = 0.0630), whereas the Log-Log plot of survival revealed a non-proportionality of the hazard [see Additional file 1 for more details on the Log-Log plot]. Since the *p*-value of the Schoenfeld residual test was close to a 5% level of significance, and the two curves crossed each other in the Log-Log Plot (i.e. indicating non-proportionality), we concluded that the Cox model was not appropriate. Thus, we resorted to a parametric determination of an AFT model. It appeared that the Weibull distribution provided the best fit for our data. We chose Weibull instead of Gompertz and Loglogistic, which are not a particular case of the generalized gamma, because the AFT model with a Weibull distribution minimized the Akaike Information Criterion (AIC).

Table 2 shows that, on average, patients treated in higher volume hospitals had a longer PFS (*p* = 0.023) than patients in lower volume hospitals.

**Table 2:** A Weibull accelerated failure time models of PFS

	Main analysis		Sensitivity analysis			
	Threshold = 12		Threshold = 5		Threshold = 8	
	Coefficient	*σ*	Coefficient	*σ*	Coefficient	*σ*
High-volume hospital	0.41***	0.136	0.21	0.141	0.33***	0.124
Age	− 0.01**	0.005	− 0.01**	0.005	− 0.01**	0.005
Cancer history	− 0.20	0.154	− v0.24	0.157	− 0.24	0.154
Ascites	− 0.32**	0.145	0.32**	0.149	− 0.34**	0.149
Neoadjuvant chemotherapy	− 0.30**	0.132	− 0.26*	0.135	− 0.29**	0.134
Histology:						
- HGSC	Ref		Ref		Ref	
- LGSC	0.23	0.490	0.28	0.499	0.30	0.495
- Mucinous	0.14	0.453	0.14	0.467	0.16	0.460
- Endometrioid	0.15	0.259	0.27	0.256	0.26	0.258
- Clear cell	− 0.01	0.279	− 0.11	0.286	− 0.16	0.284
- Unknown	− 0.19	0.209	− 0.09	0.211	− 0.16	0.209
FIGO Stage:						
- I	Ref		Ref		Ref	
- II	− 0.42	0.371	− 0.34	0.375	− 0.35	0.370
- III	− 0.58**	0.285	− 0.59**	0.292	− 0.58**	0.290
- IV	− 0.82**	0.319	− 0.85***	0.328	− 0.79***	0.324
Tumor Grade:						
- 1	Ref		Ref		Ref	
- 2	− 0.03	0.420	− 0.09	0.428	− 0.09	0.423
- 3	− 0.01	0.416	0.06	0.424	0.05	0.420
Intercept	4.77***	0.615	4.60***	0.634	4.65***	0.626

*σ*: standard deviation / Ref: modality in reference

*HGSC* High-Grade Serous Carcinoma, *LGSC* Low-Grade Serous Carcinoma

*, **, ***: significant at 10%, 5%, and 1%, respectively

We also estimated the same model with two other hospital volume activities thresholds as a sensitivity analysis. The magnitude of the coefficient associated with being treated in a HVH decreased when we employed a threshold of 8 patients treated per year, but remained strongly significant (Table 2). Whereas when we used a threshold of 5 patients treated per year there was no longer a difference in the PFS, on average, between patients treated in high- or low-volume hospitals.

### Propensity score approach: Matching using the inverse probability weighting (IPW)

Table 3 shows a good quality for the matching by IPW. Indeed, there was no significant difference for all covariates between the two groups, while there were significant differences prior to matching in terms of the stage, grade, and histology. The mean of the standardized mean differences was 7.3 for the matched sample (Table 3) compared to 20.4 for the unmatched sample.

**Table 3:** Characteristics of the patients after using IPW matching

	Low-volume hospital (*n* = 78 patients)	High-volume hospital (*n* = 189 patients)		
	m	m	*P*-value	% bias
Age	67.56	65.81	0.568	− 13.5
Cancer history	0.15	0.17	0.830	5.7
Ascites	0.69	0.67	0.920	− 2.5
Neoadjuvant chemotherapy	0.52	0.57	0.712	9.6
Histology:				
- HGSC	0.65	0.59	0.642	−11.7
- LGSC	0.05	0.03	0.700	− 8.2
- Mucinous	0.03	0.04	0.739	6.6
-Endometrioid	0.14	0.11	0.659	− 10.6
- Clear cell	0.05	0.05	0.995	0.1
- Unknown	0.08	0.17	0.318	27.5
FIGO Stage:				
- I	0.15	0.15	0.920	2.3
- II	0.08	0.07	0.916	− 2.9
- III	0.68	0.66	0.848	− 4.7
- IV	0.09	0.11	0.793	6.3
Tumor Grade:				
- 1	0.06	0.05	0.717	− 6.5
- 2	0.14	0.14	0.980	− 0.6
- 3	0.80	0.82	0.823	5.1
			*Mean:*	*7.3*
			*Median:*	*6.3*

m: mean (frequency) if the covariate is continuous (binary) / % bias, also known as the standardized difference of the mean

*HGSC* High-Grade Serous Carcinoma, *LGSC* Low-Grade Serous Carcinoma

**Fig. 2:**
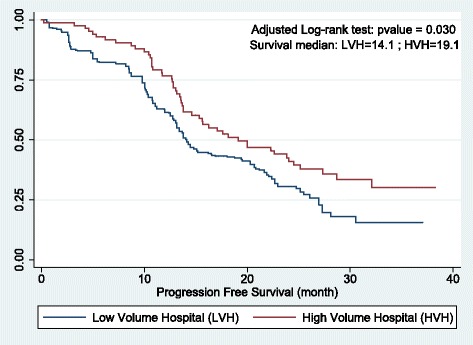
Adjusted Kaplan-Meier estimator of the Progression-Free Survival after weighing by the IPW

Matching using IPW allowed for determination of both the absolute treatment effect, with the AKME, and the relative reduction of an event occurring by the univariate weight Cox model. Figure 2, based on the AKME, indicates that patients in high-volume hospitals had a significantly longer PFS (*p* < 0.0011) than patients in low-volume hospitals. For example, the median survival for the PFS was 20 months in the high-volume hospitals, versus 14.2 months in the low-volume hospitals.

**Fig. 3:**
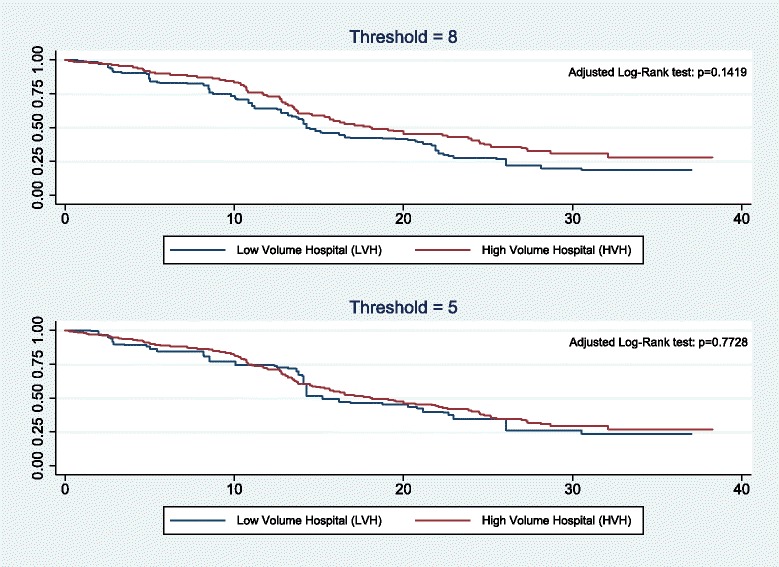
Sensitivity analysis: Adjusted Kaplan-Meier estimator of the Progression-Free Survival after weighing by the IPW with a threshold of either 5 or 8 patients treated per year and per hospital

Furthermore, the univariate Cox model of the PFS, weighted by the inverse of the propensity score, revealed that the hazard ratio (HR) (i.e. the hazard or chance of events occurring in the treatment arm as a ratio of the hazard of the events occurring in the control arm) for treatment in a high-volume hospital was HR = 0.52 (*p* < 0.001, 95% CI: [0.35; 0.75]). The Schoenfeld residual test revealed that the proportional hazard assumption was valid for the univariate weighted Cox model (*p* = 0.1410), and it confirms the robustness of the result. As a sensitivity analysis, we also ran the same analysis with a threshold of either 5 or 8 patients treated per year and per hospital. The univariate weighted Cox models revealed that the hazard ratio was HR = 0.73 (*p* = 0.082) with a threshold of 8, and HR = 0.90 (*p* = 0.632) with a threshold of 5. Figure 3, based on the AKME, indicates that there was no significant difference in survival between patients in high- and low-volume hospitals for both of the threshold of the sensitivity analysis.

## Discussion

### Definition of a high-volume hospital

In this study, we showed that being treated in a higher volume hospital increased the PFS of patients, compared to a lower volume hospital. More specifically, the probability of relapse (including death) was twice as high for patients treated in lower volume hospitals (i.e. 1.94 higher, *p* < 0.001) compared to patients treated in higher volume hospitals). Indeed, the median PFS in high-volume hospitals was 20 months, versus only 14.2 months in low-volume hospitals. Moreover, the higher proportion of complete tumor resections, and the lower proportion of reoperation (Table 1) support the notion that the quality of the first-line surgery appears to be better in high-volume hospitals, as reported previously by Ioka et al. [9] and Vernooij et al. [13].

To define a high-volume hospital, different countries have employed different thresholds that are based on the prevalence of the disease [4–14]. For example, the mean volume of activity of high-volume hospitals in the study by Ioka et al. on a Japanese dataset was 8.8 patients, which may be considered to be low compared to what has been seen with studies in the USA [9]. Yet it appears that in 2012, 93% of the hospitals had treated fewer than 12 patients in first-line treatment for EOC per year in the Rhone-Alps region of France, 82% had treated fewer than 8, and 60% had treated fewer than 5. We chose the upper quartile (12 patients) in the main analysis as the threshold, in order to obtain a share of 25% of patients treated in a HVH that is more in line with the threshold of 20 cases that is widely used in the USA, which yielded a distribution of 17.9% of patients treated in HVH in the study by Bristow et al. [6]. We also considered two other thresholds, namely 5 and 8 patients per year, as a sensitivity analysis in order to cover all of the quartiles of the patient distribution. The sensitivity analysis showed that the results were mixed when we considered a threshold of 8 cases/year, and that there was no longer a volume-outcome effect with a threshold of 5 cases/year. Indeed, with a threshold of 8 cases/year, the multivariate analysis revealed a positive impact of hospital volume activities on outcomes, whereas the propensity score analysis revealed no association at a 5% level of significance. Thus, the sensitivity analysis showed that the cut-off has to be restrictive enough in order to identify a volume outcome relationship for EOC.

Many countries already require a minimum level of activity for a hospital in order for it to be authorized to provide cancer treatments. In France, the minimum cut-off in order to receive authorization to treat gynecological cancers was defined by the French ministerial order of 27 March 2007 as 20 surgeries per year. Below this volume of activity, a hospital is no longer authorized to treat patients with gynecological cancers. This threshold, however, takes into accounts all of the various types of gynecologic cancers, such as cervical, ovarian, vaginal, uterine, and vulvar cancers. Our findings indicate that there is a need for a specific minimum activity cut-off for ovarian cancer only. Indeed, the overall threshold of 20 cases per year does not specify whether it refers to all gynecological cancers or ovarian cancer only. Out of all of the patients in first-line treatment for EOC in the Rhone-Alpes Region of France in 2012, 71% were treated in hospitals with fewer than 12 cases per year, 50% in hospitals with fewer than 8 cases per year, and 24% in hospitals with fewer than 5 cases per year. This distribution of hospital volume activities is not a specificity of the Rhone-Alpes region in France. Indeed, the public website^2^ held by the French National Authority of Health (HAS) recorded that in the most populous region of France (i.e. Ile-de-France), 118 hospitals had authorization to treat gynecologic cancers in 2017, compared with 71 for the Rhone-Alpes region. With a population of 6,574,708 for the Rhone-Alpes region and of 12,142,802 for Ile-de-France in 2016 (source: National Institute of Statistical and Economic Information), there was one hospital treating gynecologic cancers for every 92,601 residents in the Rhone-Alpes region and one for every 102,905 residents in Ile-de-France. As the number of hospitals is similar between the two regions, the distribution of hospital volume activities is also likely to be similar.

Our findings appear to support the use of a specific cut-off for ovarian cancer, and more research needs to be done for other rare cancers in order to verify whether a specific minimum activity cut-off is similarly required. Nevertheless, a threshold at the hospital level does not take into account the heterogeneity among the practitioners at any given hospital. A recent study has shown that the physician’s volume of activity also positively correlates with survival, and that the combination of being treated in a high-volume hospital by a high-volume physician appears to be superior in terms of survival compared with other combinations of hospital and physician volumes of activity [6]. More research needs to be done to develop a management program that takes into account the volume of activity at both the hospital and the physician level. Hospital participation in clinical trials has also been shown to improve EOC patient outcomes [24]. More research need to be done to properly understand what underlies the volume-outcome relationship.

### Why should we use a counterfactual approach?

We used observational data, which allowed for a better external validity than randomized controlled trials (RCT) [17]. However, in this context of observational data, which is often the case in retrospective studies analyzing the care pathway, the selection bias due to the sample heterogeneity must be taken into account [17]. Indeed, a selection bias, or recruitment bias, could appear since participation in the treatment was not random - some types of patients had a higher probability of being treated than others. Several well-known methods can be used to correct for this issue, such as stratification or multivariate analysis, and more sophistical methods are increasingly being used, such as matching using the propensity score or instrumental variable [17].

In our case, patients treated in high- versus low-volume hospitals were not similar (Table 1). Thus, we expected selection bias to occur, which means that some types of patients were more likely to be treated in a high-volume hospital than others.

The propensity score approach is based on less constrained assumptions than multivariate analysis [25, 26]. Indeed, propensity scores and multivariate analysis are based on the conditional independence assumption (CIA), which specifies that, conditional on observed covariates, patients were randomly treated in a high- or low-volume hospital. Based on the covariates recorded in our database, the CIA hypothesis assumes that two patients with the same age, cancer history, presence or not of ascites, histology, FIGO stage, neoadjuvant chemotherapy, and tumor grade will have similar outcomes (i.e. survival). However, multivariate analysis requires a stronger assumption about the distribution of the covariates and their relationship with relapse-free survival. In our case, we also had to choose a distribution of the hazard in order to fit a parametric AFT model of the relapse-free survival on a variable denoting treatment and on a set of covariates because the proportional hazard assumption was violated.

Therefore, the combination of a multivariate analysis and a matching method allowed us to determine both conditional and marginal effects of being treated in a high-volume hospital, and to prove the robustness of our findings. The conditional effect indicates that if a patient treated in a lower volume hospital was treated in a higher volume hospital, this would, on average, improve her progression-free survival (*p* < 0.001). Furthermore, the marginal treatment effect indicates that patients treated in higher volume hospitals had a probability of relapse (including death) that was nearly half that for patients treated in lower volume hospitals (1.94-fold difference, *p* < 0.001), and that the absolute difference in survival was significant (*p* < 0.001) (see Fig. 2). We have reason to be confident of the robustness of our result since both the parametric (AFT model) and the semi-parametric (propensity score) approach yielded similar results.

With both methods, the type of chemotherapy was included as an indicator denoting one if the patient received a neoadjuvant chemotherapy; without differentiating for the use of neoadjuvant alone, in combination with adjuvant chemotherapy, the use of adjuvant chemotherapy alone, or no chemotherapy at all because this study sought to measure the impact of being treated in a HVH in first-line treatment. Adjuvant chemotherapy is not a first-line treatment, however, and could hence not be included as a prognostic factor. Neoadjuvant chemotherapy has been shown to decrease the Overall Survival (OS), meaning that it is linked to observed and unobserved patient characteristics that worsen outcomes [27]. Thus, by controlling for it as a prognostic factor, we indirectly controlled for these observed and unobserved characteristics.

In the multivariate analysis, we used an AFT model instead of a semi-parametric Cox regression due to the non-proportionality of the hazard. We used the IPW matching as it was the method that best fit our data. Indeed, the IPW was the method with the lowest mean and median for the standardized difference of the mean, which indicates that this was the matching method that best balanced out the covariates between high- versus low-volume hospitals. Moreover, two simulation studies had shown that the IPW appears to perform better in determining the marginal hazard ratio of the treatment effect, compared with other matching methods [25, 28]. It should be noted that the common support of the distribution of the propensity score is sufficient [see Additional file 2] to validate the overlap assumption. The mean standardized difference in the mean before matching was 20.4 versus 7.3 after matching using the IPW, which reveals a high quality of adjustment for the IPW matching. To our knowledge, this is the first study to use a propensity score approach in regard to the question of the concentration of care in ovarian cancer, while these methods have been widely used with other diseases [29, 30].

### Limitations

Our study is based on an exhaustive regional cohort. The external validity is therefore lower compared to a national cohort. Another limitation is that we could not properly compare our results with the existing literature since we used a different threshold than the one most often used in the literature in the USA (i.e. 20 cases). We also did not control for human Breast Cancer (BRCA) gene mutations, which are known to increase the probability of developing ovarian cancer [31], co-morbidities, and being treated by a gynecological oncologist since this information was not in our database. It would have been interesting to assess the impact of the concentration of care in terms of overall survival (OS), but the OS data was not yet available.

## Conclusion

As reported in other countries, the concentration of care for EOC has a significant positive impact on patient relapse-free survival. Indeed, the results indicate that in the Rhone-Alpes region of France patients treated in lower volume hospitals had a probability of relapse (including death) that was 1.94 times higher than for patients treated in higher volume hospitals. High-volume hospitals mostly treat advanced stage EOC, while it is clear that the concentration of care improves patient survival for both advanced and early EOC. More research needs to be done on monetary and non-monetary incentives for practitioners and patients in order to promote the centralization of care for EOC in France. The above limitations should, however, not undermine the main findings of this study. The high rates of progression and death suggest that there is a pressing need for improvements in regard to EOC treatments. The centralization of care in and of itself may provide only a marginal benefit to this patient population. More importantly, centralization should provide the best opportunity to quickly and safely introduce new treatments, and to evaluate and respond to ongoing population-level outcome results.

## Additional files

Additional file 1: Log-Log progression free survival curves comparing LVH and HVH. Displays the log-log survival curves (threshold of 12 cases), which are a transformation of the standard Kaplan Meier estimator. These curves can be used to test the proportional hazard assumption. Indeed, the hazard is proportional if the two curves look parallel, meaning that the hazard ratio is constant over time. In our case, the two curves doesn’t looks parallel and even cross each other at the bottom right of the plot, meaning that the hazard is not proportional. (PDF 77 kb)

Additional file 2: Common support of the distribution of the propensity score. Displays the distribution of the propensity score for treated and untreated patients (threshold of 12 cases). The common support seems to be sufficient to allow for use of the matching method. (PDF 6 kb)

## Abbreviations

AFT: Accelerated failure time model
AKME: Adjusted Kaplan-Meier estimator
ATT: Average treatment effect on the treated
CIA: Conditional independence assumption
EOC: Epithelial ovarian carcinoma
HGSC: High-grade serous carcinoma
HR: Hazard ratio
HVH: High-volume hospital
IPW: Inverse probability of treatment weighing method
LGSC: Low-grade serous carcinoma
LVH: Low-volume hospital
OS: Overall survival
PFS: Progression-free survival
RCT: Randomized controlled trial

## Footnotes

1 With a population of 6,283,541 in 2011, the Rhone-Alpes region is the second most populous region of France. The region has several large cities; the three biggest being Lyon, Grenoble, and Saint-Etienne. The Rhone-Alpes region is located in the southeast of France, and it merged with the Auvergne region to form the Auvergne-Rhone-Alpes region in 2016. However, since patients in the database were treated in 2012, we will refer to the Rhone-Alpes region only, as it was delimited in 2012.

2
https://scopesante.fr
