# Supplementary material for: Being treated in higher volume hospitals leads to longer progression-free survival for epithelial ovarian carcinoma patients in the Rhone-Alpes region of France
Source: BMC Health Serv Res. 2018 Jan 4;18:3. doi: 10.1186/s12913-017-2802-2 (PMC5755403; doi:10.1186/s12913-017-2802-2)
Supplement: Supplementary file 1 — Log-Log progression free survival curves comparing LVH and HVH. Displays the log-log survival curves (threshold of 12 cases), which are a transformation of the standard Kaplan Meier estimator. These curves can be used to test the proportional hazard assumption. Indeed, the hazard is proportional if the two curves look parallel, meaning that the hazard ratio is constant over time. In our case, the two curves doesn’t looks parallel and even cross each other at the bottom right of the plot, meaning that the hazard is not proportional. (PDF 77 kb) [file 12913_2017_2802_MOESM1_ESM.pdf]

Additional file 1 displays the log-log survival curves (threshold of 12 cases), which are a transformation of the standard Kaplan Meier estimator. These curves can be used to test the proportional hazard assumption. Indeed, the hazard is proportional if the two curves look parallel, meaning that the hazard ratio is constant over time. In our case, the two curves doesn't looks parallel and even cross each other at the bottom right of the plot, meaning that the hazard is not proportional.

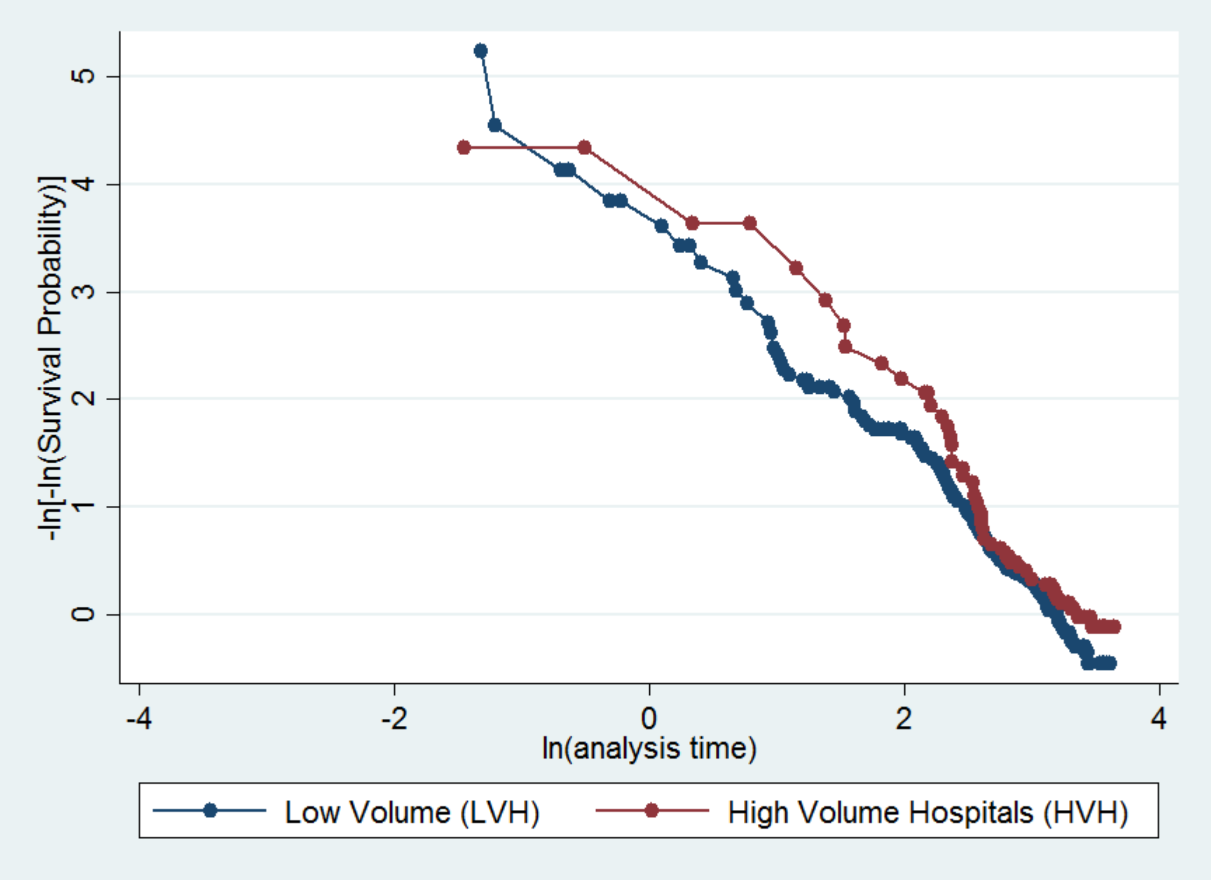

Additional file 1: Log-Log progression free survival curves comparing LVH and HVH
