# Supplementary material for: Being treated in higher volume hospitals leads to longer progression-free survival for epithelial ovarian carcinoma patients in the Rhone-Alpes region of France
Source: BMC Health Serv Res. 2018 Jan 4;18:3. doi: 10.1186/s12913-017-2802-2 (PMC5755403; doi:10.1186/s12913-017-2802-2)
Supplement: Supplementary file 2 — Common support of the distribution of the propensity score. Displays the distribution of the propensity score for treated and untreated patients (threshold of 12 cases). The common support seems to be sufficient to allow for use of the matching method. (PDF 6 kb) [file 12913_2017_2802_MOESM2_ESM.pdf]

Additional file 2 displays the distribution of the propensity score for treated and untreated patients (threshold of 12 cases). The common support seems to be sufficient to allow for use of the matching method.

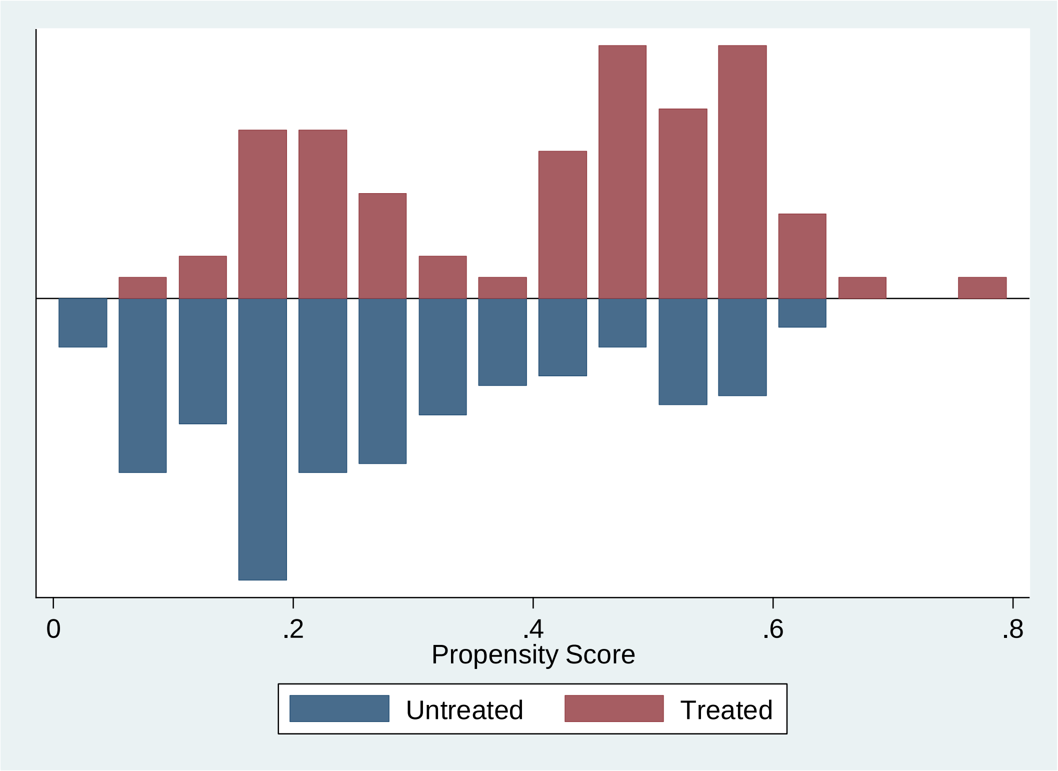

Additional file 2: Common support of the distribution of the propensity score
